# Small but significant: Insights and new perspectives of exosomes in cardiovascular disease

**DOI:** 10.1111/jcmm.15492

**Published:** 2020-06-24

**Authors:** Jianchao Zhang, Xiaolin Cui, Jiacheng Guo, Chang Cao, Zenglei Zhang, Bo Wang, Li Zhang, Deliang Shen, Khoon Lim, Tim Woodfield, Junnan Tang, Jinying Zhang

**Affiliations:** ^1^ Department of Cardiology The First Affiliated Hospital of Zhengzhou University Zhengzhou Henan China; ^2^ Henan Province Key Laboratory of Cardiac Injury and Repair Zhengzhou Henan China; ^3^ Department of Orthopaedic Surgery & Musculoskeletal Medicine University of Otago Christchurch New Zealand; ^4^ Medical Technologies Center of Research Excellence Christchurch New Zealand

**Keywords:** cardiovascular disease, cellular communication, diagnostic, exosomes, therapy

## Abstract

Cardiovascular diseases (CVDs) are a major health problem worldwide, and health professionals are still actively seeking new and effective approaches for CVDs treatment. Presently, extracellular vesicles, particularly exosomes, have gained its popularity for CVDs treatment because of their function as messengers for inter‐ and extra‐cellular communications to promote cellular functions in cardiovascular system. However, as a newly developed field, researchers are still trying to fully understand the role of exosomes, and their mechanism in mediating cardiac repair process. Therefore, a comprehensive review of this topic can be timely and favourable. In this review, we summarized the basic biogenesis and characterization of exosomes and then further extended the focus on the circulating exosomes in cellular communication and stem cell‐derived exosomes in cardiac disease treatment. In addition, we covered interactions between the heart and other organs through exosomes, leading to the diagnostic characteristics of exosomes in CVDs. Future perspectives and limitations of exosomes in CVDs were also discussed with a special focus on exploring the potential delivery routes, targeting the injured tissue and engineering novel exosomes, as well as its potential as one novel target in the metabolism‐related puzzle.

## 1 INTRODUCTION

Cardiovascular diseases (CVDs) are a major health problem worldwide. According to the Centers for Disease Control and Prevention, CVDs are the primary cause of death in the United States.^1^ The ischaemic heart disease, one of major contributors to CVDs, has been the leading cause of death at the national level in China in 2017.^2^ The treatments for CVDs have been intensively investigated including stem cells therapy and its derived growth factor‐based therapy.^3^, ^4^ However, the inherent limitations of those therapies, such as severe immune rejection, low stability in shipping and storage, short half‐life, result in the unsatisfied clinical outcome.^5^ Therefore, there is a high demand for developing new therapies in CVDs treatment. Recently, the utilization of paracrine principle to regenerate ischaemic tissues by mediating cellular communications for CVDs treatment has gained its popularity, due to its effectiveness of recovering cardiac function after the initial infarction.^5^ Among those paracrine signalling factors playing a role in cardiac repair, extracellular vesicles (EVs) specifically draw increasing interest because of their ability in regulation of cellular communication resulting in the promotion of cellular function.

Extracellular vesicles were first considered as cellular membrane debris without biological significance, until Rapose reported that EVs played a role in the simulation of immune response.^6^ Since then, studies have shown the importance of EVs in cellular communication, and the identification of EVs gained growing interests. Both microvesicles and apoptotic bodies were identified as EVs that play a role in the biological processes.^7^, ^8^ In 1983, another small‐sized EVs were discovered first time in rat reticulocytes and later were named as exosomes by Raposo et al^9^ Initially, exosomes were considered cell ‘dumpsters’ that contain undesirable cellular waste to maintain cellular homeostasis. Later studies indicated that exosomes are involved in intercellular connection^10^ and play an important assignment in multiple physiological and pathological processes.^11^ As a result, exosomes, as a biocomponent, have been intensively investigated.

**FIGURE 1:**
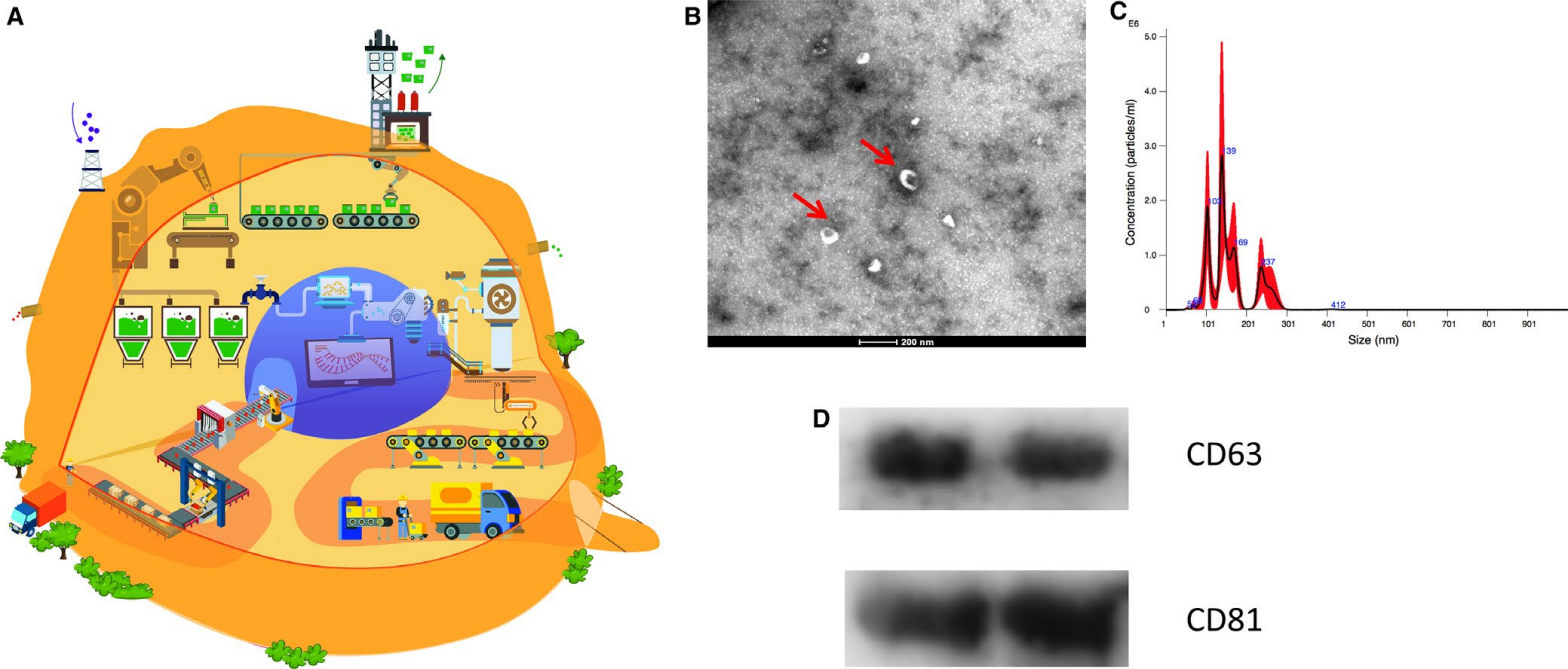
Schematic image for cell secreted or circulating exosomes. A, A cell works like a factory secreting factors and extracellular vesicles, and exosomes are secreted like packages with specific proteins and exRNAs. B, TEM analysis on circulating exosomes(red arrows indicating exosomes). Scale bar = 200 nm. C, The distribution on size of exosomes analysed by Nanoparticle tracking analysis (NTA). D, Western blotting analysis on the surface makers as CD63 and CD81 of exosomes

The exosome is typically classified as an EV with a diameter of 40‐150 nm,^12^ which are endosome‐derived, and secreted by most, if not all, cells. They normally contain lipids, proteins and various RNA species (including mRNA, miRNAs and lncRNAs),^13^ depending on the cell type and the cellular microenvironment (Figure 1). Additionally, the exosomes released by the same cell line can be heterogeneous due to the different cargo‐sorting mechanisms. Through releasing the proteins and nucleic acids, exosomes can mediate local as well as systemic cell‐to‐cell communications by intervening with cellular physiological change. However, the mechanisms of the exosome‐stimulated signalling pathway remain unclear. A previous study indicated that this process involves receptor‐ligand interactions and exosomal internalization, resulting in the release of cargo into the cytoplasm of recipient cells.^14^

Because of the vital biocomponent with exosome contributing to intercellular and extracellular communications, scientists have been postulating if the exosome may mediate pathological pathways in cardiac tissue. Experiments conducted by Barile^15^ and Ibrahim^16^ showed that cardiac progenitor cells (CPCs) and human cardiospheres (CSp) generated EVs with enriched anti‐apoptotic and proangiogenic miRNAs such as miR‐210, miR‐132 as well as miR‐146a, which can up‐regulate angiogenesis of endothelial cells and recover left ventricle (LV) function of the post‐ischaemic heart. Similar studies used mouse CPCs and fibroblast‐induced pluripotent stem cells (iPSC) to isolate exosomes for cardioprotection and anti‐apoptosis. Another study extracted exosomes from mouse embryonic stem cells (ESCs) and identified that miR‐294 is the main driven force to augment CPCs and cardiomyocytes proliferation‐based endogenous cardiac repair.^17^ All those studies suggest that exosomes hold a great clinical potential in CVDs treatment.

However, despite significant research attention and developments in exosomes and their potential applications in CVDs treatment, there is a lack of an informative summary on therapeutic and diagnostic functions of exosomes in CVDs, especially the inter/extra‐cellular communication between the cardiovascular system and other organs mediated through exosomes. The aim of this review is to provide a detailed overlook of the exosomes and their roles in cellular communication, which lead to their ability in diagnosing and treating CVDs. First, an overview of the fundamental biogenesis and characterization of exosome is discussed. Next, we describe the role of exosome in cardiac tissue communication in details with a special focus on stem cell‐derived exosomes in infarcted heart, which provides the underlined working mechanism of exosome. We then review the interaction between heart and distant organs through exosomes, together with the diagnostic and therapeutic role of exosomes in CVDs using the most recent studies to showcase the progression of exosome‐based therapy in current clinical applications. Finally, future perspectives and current limitations of exosome in CVDs treatment are discussed. We believe that there is much progress that can be made as the understanding of exosomes are further advanced and hope that this review—by introducing the mechanism of exosome‐mediated cellular communication within cardiac tissue or through circulatory system—will help to promote the development exosome‐based therapy in CVDs diagnosis and treatment.

## 2 BIOGENESIS AND CHARACTERIZATION OF EXOSOMES

### 2.1 Biogenesis of exosomes

As nano‐sized particles, exosomes carry many vital biocomponents that mediate their function and represent a unique class of EVs by virtue of their biogenesis. The biogenesis of exosomes involves different steps. Early endosomes must mature into late endosomes or multivesicular bodies (MVBs); then, the invagination of the plasma membrane allows for the generation of intraluminal vesicles (ILVs) in the lumen of organelles.^18^ MVBs further fuse with the plasma membrane to release ILVs into the extracellular matrix; the MVBs are called exosomes at this stage. Rab GTPases 27A and 27B are reported to be the two components that can mediate the fusion and docking of MVBs to the plasma membrane, thus promoting exosome production.^19^

The process of sorting cargo within exosomes involves endosomal sorting complexes required for transport (ESCRT) and tetraspanin‐ and lipid‐dependent mechanisms.^20^ ESCRT‐0, ESCRT‐I, ESCRT‐II and ESCRT‐III protein complexes play roles on producing the ILVs that bud into MVBs and in sorting monoubiquitinated proteins via an ESCRT‐dependent mechanism. For example, the ESCRT‐III‐associated protein ALIX can affect cargo loading and MVB subtypes.^21^ The protein Hrs within ESCRT‐0 is involved in exosome secretion.^22^ Tetraspanins such as CD82, CD9 and CD63 are factors involved in the ESCRT‐independent process of sorting exosome cargo.^23^ Studies have indicated that CD9 and CD82 can up‐regulate the exosomal release of β‐catenin from HEK293 cells.^24^ Furthermore, the knockout of CD63 can reduce the secretion of EVs, which proves the key role of CD63 in the exosome biogenesis process.^25^ A recent study suggested that two cargo‐sorting pathways might simultaneously happen, resulting in different subpopulations of exosomes depending on the different machineries.^26^ Besides, cell type and cellular homeostasis are other factors found to control the secretion of exosomes.

The components of exosome cargo include proteins, lipids and various RNA species. Simons et al^27^ reported that exosomes convey abundant proteins, including integrins, tetraspanins, flotillins and Rab GTPases, depending on their endosomal origin. Moreover, the resulting protein profiles of exosomes from the same cell type can be discriminated, and these differences are always dependent on the microenvironment and the physiological states of the parent cells.^28^ Tetraspanins (CD9, CD63 and CD81), 14‐3‐3 proteins, heat shock proteins (HSPs), Tsg101 and the ESCRT‐3‐binding protein Alix, which are abundant in EVs, could be used as specific markers.^29^ Tetraspanins were previously considered as specific markers for exosomes, but these proteins could be found in apoptotic bodies and microvesicles.^30^ Additionally, cellular signalling proteins such as β‐catenin, TNFα, Wnt5, TGF‐β, delta‐like 4 and Notch ligand can be found within exosomes. Cytoskeletal and metabolic proteins (glyceraldehyde 3‐phosphate dehydrogenase, GAPDH) have also been identified in cargo.^31^ Interestingly, Jeppesen et al^32^ found that classical exosomes bearing tetraspanin exosomal markers (CD9, CD63 and CD81) lack luminal proteins such as GAPDH, enolase and HSP90. Cytoskeletal proteins are also not present in classic exosomes. The membrane‐bound annexins A1 and A2 are markers of non‐exosomal small EVs but not of exosomes. Specifically, extracellular double‐stranded DNA is not associated with classical exosomes.

Apart from proteins, lipids are critical in the vesicular transportation; however, the lipid component in the cargo is still unclear.^33^ Some lipid molecules that participate in exosome biogenesis, such as lysobisphosphatidic acid, have been found in exosomes.^34^ Additional lipid raft molecules, such as cholesterol, sphingolipid ceramide and glycerophospholipids, are also found in the cargo. Furthermore, lipid molecules that can mediate cellular signalling pathways, such as prostaglandins, rearrange within exosomes.^35^

The most important components of exosome cargo are cell type‐specific mRNAs and miRNAs. miRNAs are well known to play a crucial role in the regulation of multiple biological processes. As a result, those small non‐coding RNA molecules encapsulated within exosomes can mediate cell‐to‐cell communication, which suggests potential subsequent bioapplications. Exosome‐encased miRNAs, such as miR‐292, miR‐20a, miR‐17 and miR‐22, are involved in CVDs, and miR‐21 is abundant in cardiac fibroblast‐derived exosomes.^36^ These miRNAs have important roles in cell signalling in cardiac tissue. Interestingly, one quantitative and stoichiometric approach was applied to analyse the miRNA content in exosomes regardless of the source; the results indicated that most individual exosomes in standard preparations do not carry biologically significant numbers of miRNAs.^37^ Hence, the accurate role of miRNAs in exosomes needs to be further exploited. Compared to miRNAs, lncRNAs are more tissue‐specific and have been identified as critical mediators of cardiac remodelling and valuable diagnostic markers. For example, the exosomal lncRNAs could take part in the mediating of ageing‐induced cardiac dysfunction.

### 2.2 Isolation and characterization of exosome

Traditional exosome isolation methods comprise ultracentrifugation, density sucrose or iodixanol gradient centrifugation, precipitation, immune‐affinity capture and so on. However, the distinctions among different EV subgroups need to be standardized resulting from the slight diversity in physical properties and composition. Thus, although the specific subtype of EVs known as exosomes has attracted a large amount of attention, the definitive characterization of exosomes has proven to be elusive due to the heterogeneity of EV species and the assortment of non‐specific isolation techniques.^12^ Notably, Jeppesen et al^32^ recently reported a more accurate method for exosome extraction that used high‐resolution density gradient fractionation to separate small EVs from non‐vesicular material and direct immunoaffinity capture to specifically isolate exosomes from other types of small EVs.

The characterization of exosomes has been a challenge due to their nano‐scale size. Additional creative and advanced approaches need to be applied to characterize exosomes. Antibody‐based methods are popular, whereby proteins such as CD9, CD63 and CD81 can be applied as specific markers because of the endosomal pathway. Additionally, the expression of phosphatidylserine in exosomes allows other methods to also be used to characterize exosome populations.^38^ Transmission electron microscopy (TEM) enables the visualization of exosomes, and double membrane‐bound vesicles have been observed to have a ‘cup‐shape’, which is defined as the specific morphology of exosomes under TEM.^39^ Nanoparticle tracking analysis (NTA) can detect nanoparticles ranging from 50‐1000 nm and visualize individual particles by analysing the properties of light scattering and Brownian motion. Alternative options such as dynamic light scattering based on Brownian motion and LZON qNano based on tuneable resistive pulse sensing can also measure the size of exosomes. However, all of these techniques are unable to identify exosomes among all vesicles. Flow cytometer may be able to detect the existence of exosome but fails to quantify the particles because of swarming effects.^40^ Other techniques, such as atomic force microscopy,^41^ Raman microspectroscopy,^42^ small‐angle X‐ray scattering^43^ and field emission scanning electron microscopy,^41^ have been also applied to detect exosomes. To date, it is widely accepted that isolated exosomes need to be characterized for their specific markers (CD9, CD63, CD81), their sizes (NTA) along with their morphologies (TEM) together in order to confirm their identity.

## 3 THE COMMUNICATIVE ROLE OF EXOSOMES

### 3.1 Exosomes in cardiac cell‐cell communication

**FIGURE 2:**
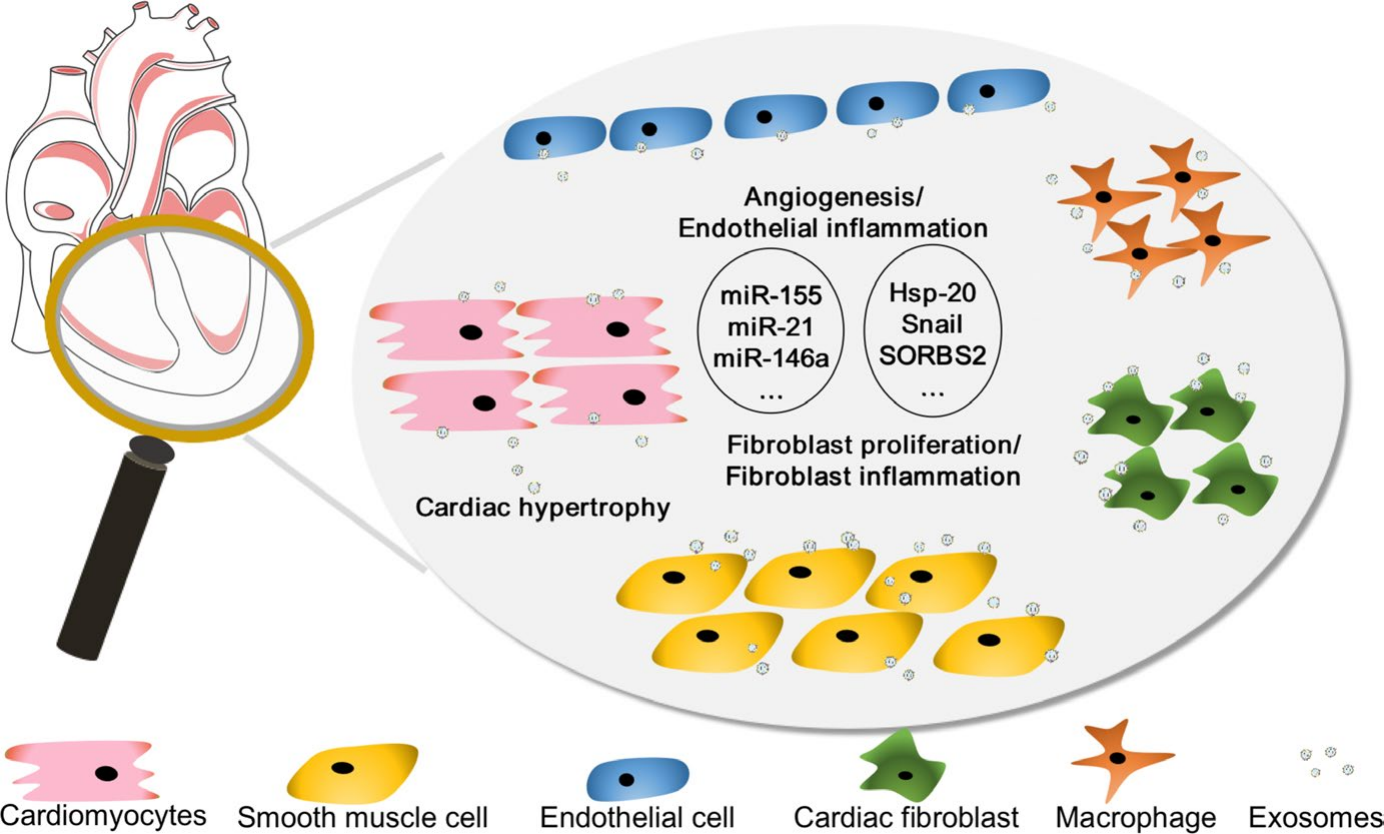
Exosomes mediated effect on cardiac microenvironment. Cardiac‐related exosomes could have several effects on recipient cells. At the original cardiac tissue site, the microenvironment could be regulated by the complex interaction with exosomes derived from surrounding cells, including cardiomyocytes, macrophages, cardiac fibroblasts and other cardiac cells. The secretion of exosomes (exRNAs, proteins, etc) may directly participate in extracellular matrix (ECM) remodelling involved in the inflammatory process in the local area, cell apoptosis, fibroblasts differentiation, cardiac hypertrophy, new vessel formation and so on

The production of exosomes allows cells to communicate by interacting with or taking up exosomes from other cells in cardiac tissue (Figure 2), given the fact that the various RNA species, lipidS and proteins within exosomes can involve in the transcription and translation process resulting in the regulation of cellular proliferation and function.^44^ In cardiac tissue, studies have shown that exosomes can mediate the communication between endothelial cells, smooth muscle cells, cardiomyocytes, monocytes, dendritic cells and fibroblasts.^45^, ^46^ For example, activated macrophage‐derived exosomes containing miR‐155 have been shown to decrease the fibroblast proliferation and promote fibroblasts inflammation, when cardiac tissue is injured.^47^ In addition, exosomes derived from mature dendritic cells increase endothelial inflammation and atherosclerosis via the membrane TNF‐α‐mediated NF‐kB pathway.^48^ Other evidence showed that the crosstalk between cardiomyocytes and endothelial cells involves cardiac exosomes. HIF‐1α can be found in the exosomes released by cardiomyocytes under hypoxic conditions; this protein can up‐regulate the expression of Hsp20^49^ and promote angiogenesis by increasing the expression of vascular endothelial growth factor receptor‐2 in endothelial cells.^50^ Another finding showed that cardiac fibroblast‐derived exosomes contain abundant miRNA fragments (such as miR‐21*) that are normally eradicated during the miRNA biogenesis process can induce cardiomyocyte hypertrophy to achieve the crosstalk between cardiac fibroblasts and cardiomyocytes.^10^

Acute myocardial infarction (AMI) leads the pathological alteration within the cardiac tissue, resulting in the changes in the number and cargo of exosomes. A recent study showed the numbers of MVBs and exosomes increased after AMI,^51^ and their actions were likely exerted by mediating cell‐cell signalling via the transport of bioactive proteins between cells,^52^ resulting in the direct activation of target cells.^53^, ^54^ Additionally, these miRNAs and proteins delivered by circulating EVs derived from red blood cells, platelets and white blood cells can mediate the activation of plaque macrophage through vascular cell adhesion molecule‐1 (VCAM‐1) inhibition during atherosclerosis progression.^55^ The transcription factor, Snail within the exosomes released after the infarction, could induce endothelial‐to‐mesenchymal transition, which aggregates the process of fibrosis formation.^56^ In another study, Qiao et al^57^ showed that exosomes derived from heart failure patients had miR‐215p dysregulation resulting in the impairment of the regenerative potential, which could further showcase the pathological changes within the cardiac tissue altering the miRNA cargo and cellular function. Apart from the role in mediating the pathological changes after infarction, exosomes can also active cardiac protections via cellular communication. For example, compared with exosomes from fibroblasts, exosomes from cardiosphere‐derived cells (CDCs) are enriched in miR‐146a, miR‐181b and miR‐126. In a rat AMI model, the exosomal transfer of miR‐181b from CDCs to macrophages could reduce the transcript level of PKCδ and decrease the transcript levels of E‐selectin, VCAM‐1 and intercellular adhesion molecule‐1 (ICAM‐1) in endothelial cells, that play an important role in the cardioprotective effects that is similar to CDCs.^58^ Apart from the direct involves in the communication between cells, exosomes can also indirectly regulate the performation of cells and subsequently affect the progression of cardiac disease. For example, the Y RNA fragment in the EVs, including exosomes, secreted by CDCs could alter IL‐10 gene expression and enhance IL‐10 protein secretion in macrophages, which were potently cytoprotective in oxidative stressed cardiomyocytes.^59^

Therefore, exosomes play important roles in the cellular communication within cardiac tissue, which can be potentially utilized for CVDs diagnosis and treatment.

### 3.2 Interplay between the heart and distant organs through exosomes

**FIGURE 3:**
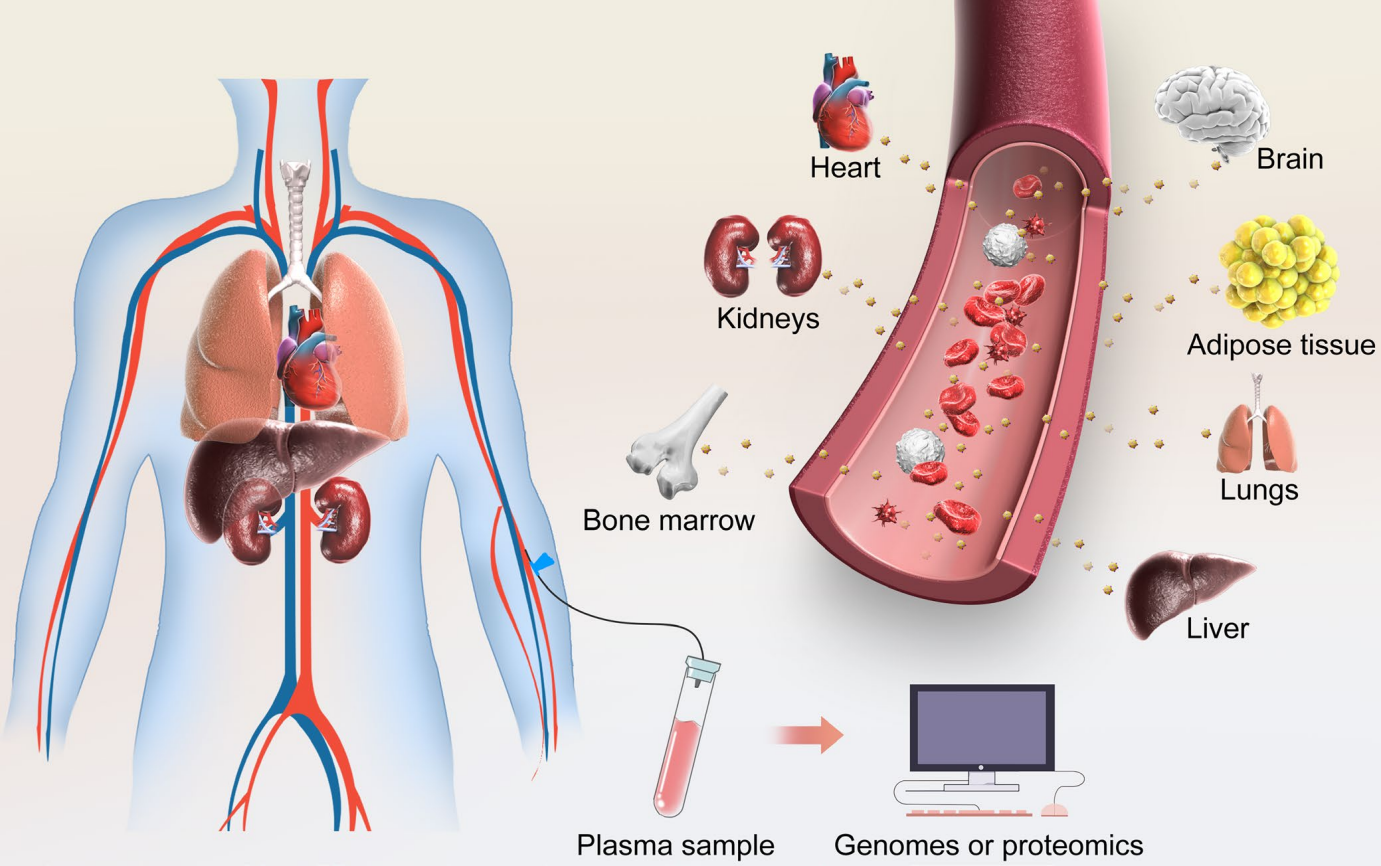
Exosomes could mediate biological effect at distant sites. Exosomes secreted from multiple cells and inherit the substance (i.e. exRNA, proteins) from cells, further being released into circulation, travelling to distant sites and producing biological function

In addition to their role in cell‐cell communication, exosomes play a role in interaction between cardiac organs and distant organs (Figure 3). There is an evidence indicating an active role for exosomes in the communication between the heart and bone marrow. For example, myocardial miRNAs could be delivered by cardiac exosomes and recruit circulating progenitor cells into an infarcted area for cardiac repair, which indicates that exosomes may mediate the functional crosstalk between ischaemic heart and bone marrow.^60^ Apart from bone marrow, Oikonomou et al^61^ suggested that the interplay between adipose tissue and the cardiovascular system was bidirectional, with vascular‐derived and heart‐derived signals directly affecting the biology of adipose tissue. Visceral adipose tissue‐derived exosomes from mice with high‐fat diet‐induced obesity could promote M1 pro‐inflammatory polarization of macrophages and further accelerate atherosclerosis in APOE^−/−^ mice.^62^ The cardiac‐specific overexpression of Hsp20 remarkably attenuated diabetes‐induced cardiac dysfunction and adverse remodelling by modulating cardiomyocyte exosome secretion.^63^

Thus, the function of cardiac tissue and the secretome is tightly controlled by complex homeostatic mechanisms, and the exosomes circulated within the circulatory systems can medicate the communication between the heart and other organs. However, the detailed molecular mechanism in the interaction between the heart and other organs remain elusive, and approaches to accurately define the source of exosomes in circulation need to be addressed. Nevertheless, the comprehensive understanding of the exosome function in communications between cardiac tissue and other tissues may hold the potentials in early diagnosis for different diseases in clinical setting.

## 4 EXOSOMES AS DIAGNOSTIC TOOLS IN CARDIOVASCULAR DISEASES

Since pathological changes to the cardiac tissue after CVDs can alter the content of exosomes, Sluijter et al^64^ proposed that exosomes from different sources may be useful biomarkers to diagnose various CVDs. Given the cell types and status of the original cells decide the contents of exosome (ie miRNAs, proteins, IncRNAs), exosomes are widely generated and derived from multiply types of cells and actively participate in a wide range of cardiovascular processes, both physiological and pathological.^65^ A significant amount of evidence has shown that exosomes seem to be associated with myocardial ischaemia and that exosome levels correlate with the severity of myocardial injury. In hypoxic or ischaemic environments, large numbers of exosomes with unique miRNA are released into plasma; thus, exosome levels are elevated in patients with CVDs or AMI.^64^ In one study, the expression of circulating exosomal miR‐133a originating mainly from infarcted and peri‐infarcted myocardium was significantly elevated in patients with acute coronary syndrome. Furthermore, the level of serum miR‐133a was elevated within 2 hours after the onset of chest pain, before creatine kinase and troponin were increased.^66^ In addition, Cheow et al^67^ identified six novel proteins located in plasma‐derived EVs that are considered as potential biomarkers of myocardial injury; these reflected post‐infarct pathways of complement activation (C1Q1A and C5), lipid metabolism (APOD and APOC3) and platelet activation pathways (GP1BA and PPBP). Some of these miRNAs may be transported by EVs, especially under pathological conditions.^68^ Thus, the number and contents of exosomes are considered early and disease‐specific biomarkers for CVDs.^69^ The determination of exosomal contents is crucial for clinicians to rapidly diagnose and identify diseases, prevent disease progression and improve prognosis.

Apart from their ability to reflect the physiological and pathological alteration within the cardiac tissue,^70^, ^71^ exosomes have the ability to protect the substance (ie miRNAs, proteins, IncRNAs) inside from RNAase to achieve more feasible and accurate diagnostic outcome. For example, various circulating miRNA including miR92a/b, miR1, miR499, miR133, miR122 is overexpressed in CVDs patients.^72^, ^73^ However, miRNAs or IncRNAs alone are not stable in circulation and easily disintegrated by enzymes. Thus, genomes or proteomics analysis on exosome profiles could be more accurate and comprehensive. Moreover, exosomes are easily harvested and distributed widely. Body fluids, including blood, urine, plasma and semen, have been proven to contain exosomes, indicating the practicality of using exosomes in current clinical settings.^74^ Taken together, exosomes have superior advantages as a tool for the diagnosis in CVDs.

## 5 EXOSOMES AS THERAPEUTICS IN CARDIOVASCULAR DISEASES

In addition to their capability to reflect early pathological changes working as a diagnostic tool, exosomes can also contribute to the cardiac tissue repair because of their ability to intervene the physiological and pathological process of cells. Often, after myocardial injury, the loss of cardiomyocytes cannot be rescued in current clinical setting. Recently, considerable effort has been made to develop cell‐based cardiac repair therapies aiming to promote cardiomyocytes proliferation and reactivation.^75^ However, low survival rate of transplanted cells, limited differentiation capacity into new functional cardiomyocytes, immune rejection and other factors limit the clinical application of stem cell therapy.^5^ Increasing evidence has indicated that exosomes derived from stem cells can play an important role in cardiac repair. Studies showed that stem cells mediate cardioprotection through autocrine and paracrine factors. Given the contents of exosomes including enriched miRNAs, growth factors, lipids and proteins, exosomes can be employed to mediate the onsite proliferation and activation of cardiomyoctes resulting in the regeneration of infarcted tissue.^76^, ^77^ Furthermore, exosomes possess ability to bypass the phagocytosis and the engulfment by lysosomes with low immune responses, resulting in an improved therapeutic effect.^78^, ^79^ Exosomes derived from different cell sources have been reported to promote a similar level of cardioprotection to their parent cells in preclinical experiments ^80^, ^81^ as shown in Table 1. Exosomes used for CVDs treatment can be divided into two categories based on their parent cells source including cardiac resident cells and stem cells.

**Table 1:** Summary on the therapeutic function of exosomes derived from stem cell in heart ischaemic model

Cell source	Disease models	Injection method	Contents	Involve pathways	Biological effects	Reference
Mouse ESCs	Mouse MI model	Intramyocardial injection	miR 290‐295 cluster	Undefined	Stimulates and augments CPC and cardiomyocyte proliferation	^17^
Hypoxia‐conditioned BM‐MSCs	Mouse MI model	Intramyocardial and intravenous injection	miR125b‐5p	Suppressing *p53* and *BAK1*	Facilitates ischaemic cardiac repair by ameliorating cardiomyocyte apoptosis	^80^
Human ESC‐MSCs	Mouse myocardial I/R model	Intravenous injection	Whole content	Undefined	Decreases infarct size	^81^
Human CDCs	Pig acute and chronic MI model	Intracoronary and intramyocardial injection	Whole content	Undefined	Decreases scarring, attenuates adverse remodelling and improves cardiac function after MI	^82^
Human CDCs	Pig chronic MI model	Intramyocardial injection	Whole content	Undefined	Preserves cardiac function and reduces scar size	^83^
Mouse BM‐MSCs	Mouse MI model	Intramyocardial injection	Whole content	Undefined	Stimulates neovascularization, restrains inflammation response and preserves cardiac function	^92^
MSCs	Mouse myocardial I/R model	Intramyocardial injection	miR‐182	Targeting TLR4/NF‐κB/PI3K/Akt signalling cascades	Reduces infarct size and alleviates cardiac inflammation	^93^
Mouse BM‐MSCs	Mouse MI model	Intrapericardial injection	miR‐21a‐5p	Down‐regulating expression of the pro‐apoptotic gene products PDCD4, PTEN, Peli1 and FasL	Decreases infarct size	^108^

Abbreviations: BM, bone marrow; CDCs, cardiosphere‐derived cells; CPC, cardiac progenitor cell; ESCs, embryonic stem cells; I/R, ischaemia/reperfusion; MI, myocardial infarction; MSCs, mesenchymal stromal cells.

### 5.1 Cardiac resident cells derived exosome

Cardiac resident cells include CPC, Sca‐1 + CPCs and side population cells. Given the fact that those cells have been reported to contribute to the cardiac repair, scientists have explored the possibility of using their derived exosomes in CVDs treatment. For example, Gallet et al^82^ performed a randomized placebo‐controlled study in a pig model of convalescent MI and suggested that exosomes from CDCs could effectively attenuate adverse remodelling, improve cardiac function and suppress scarring. Furthermore, Nguyen et al^83^ used the diffusion tensor cardiac magnetic resonance (DT‐CMR) technique and found that exosomes from CDCs could play a vital role in preserving myocardial fibre architecture, reducing scar size and attenuating adverse remodelling. Additionally, Ibrahim et al^16^ demonstrated that EVs from CSp could restore cardiac function by decreasing cell apoptosis and promote new vessel formation due to the enriched content of anti‐apoptotic and proangiogenic miRNAs, namely miR‐210, miR‐132 and miR‐146a, within EVs. Apart from CSp, CPC‐derived exosome has also been used for myocardial infarction treatment, in which CPC‐derived exosomes can inhibit cardiomyocytes apoptosis and improve cardiac function.^15^ A recent report has demonstrated exosome derived from CXCR4‐overexpressing CPCs might improve heart function by transferring exogenous proteins and mRNA to the target cells.^84^ In another study, pregnancy‐associated plasma protein‐A (PAPP‐A, also known as pappalysin‐1) plays a key role in CPCs exosome‐mediated cardioprotection by protolytic cleaving insulin‐like growth factor‐binding protein to promote the release of insulin‐like growth factor‐1, which active the intercellular ERK1/2 and Akt.^85^

### 5.2 Stem cell‐derived exosome

Similarly, the therapeutic effects of exosome derived from iPSCs and iPSC‐derived cardiomyocytes (iCMs) have been regarded as one possible opportunity to repair damaged tissue and restore cardiac function. Jung and colleagues summarized that exosomes derived from iCMs inherit the protective molecules to salvage the injured heart.^86^ Importantly, some researchers have investigated that EVs derived from iPSCs are safer and more effective for cardiac function preservation than cells themselves.^87^ Lai et al^81^ found that mesenchymal stromal cell (MSC)‐derived exosomes have the cardioprotective effect of reducing infarct size in a mouse ischaemia/reperfusion model. Mayourian et al^88^ revealed that miR‐21‐5p plays a key role in exosomes derived from human MSCs, increasing cardiac tissue contractility and calcium handling via PI3K signalling. It has also been demonstrated that RNAs and miRNAs in the supernatant of human MSCs could have a cardioprotective effect in a rat model of ischaemia/reperfusion, which had potential affinity with exosomes.^89^ Cardioprotective effects were also found in bone marrow stem cells secretome. For instance, Sahoo et al^90^ found that exosomes secreted by CD34^+^ stem cells could promote vessel formation. Additionally, exosomes from CD34^+^ stem cell are further confirmed to be involved in the transfer of miR‐126‐3p, which up‐regulated the expression of angiogenic genes, such as VEGF and ANG1.^91^ Therefore, exosomes and exosomal contents (mRNA, miRNA and other RNAs) derived from MSCs,^92^, ^93^, ^94^ iPSCs,^86^, ^87^ ESCs^17^ and even pericardial fluid ^95^ have been identified the effect on protecting heart by stimulating proangiogenic, proliferative, anti‐apoptotic and anti‐inflammatory signalling cascades in CVDs. More recent study conducted by Biemmi et al has shown that circulating inflammatory EVs significantly increase after the infarction and they can activate TRL4‐dependent NF‐ĸB inducing cell death. By reducing the number of inflammatory EVs during the acute phase of ischaemia, left ventricular ejection fraction can be preserved.^96^ Taken together, exosomes can be one of the key solutions for CVDs treatment in the future.^97^, ^98^

## 6 DISCUSSION AND FUTURE PERSPECTIVES

Exosomes have gained considerable interest in CVDs diagnosis and treatment due to their ability to reflect the physiological and pathological alteration within cardiac tissue as well as capability to mediate cellular communication to promote tissue repair. With increasingly global popularity in exosomes, scientists have gain more comprehensive knowledge and understanding in the role of exosomes playing in the cellular communication. However, there are certain challenges that need to be overcome before the exosome‐based therapy can rapidly progress into clinical trials.

**FIGURE 4:**
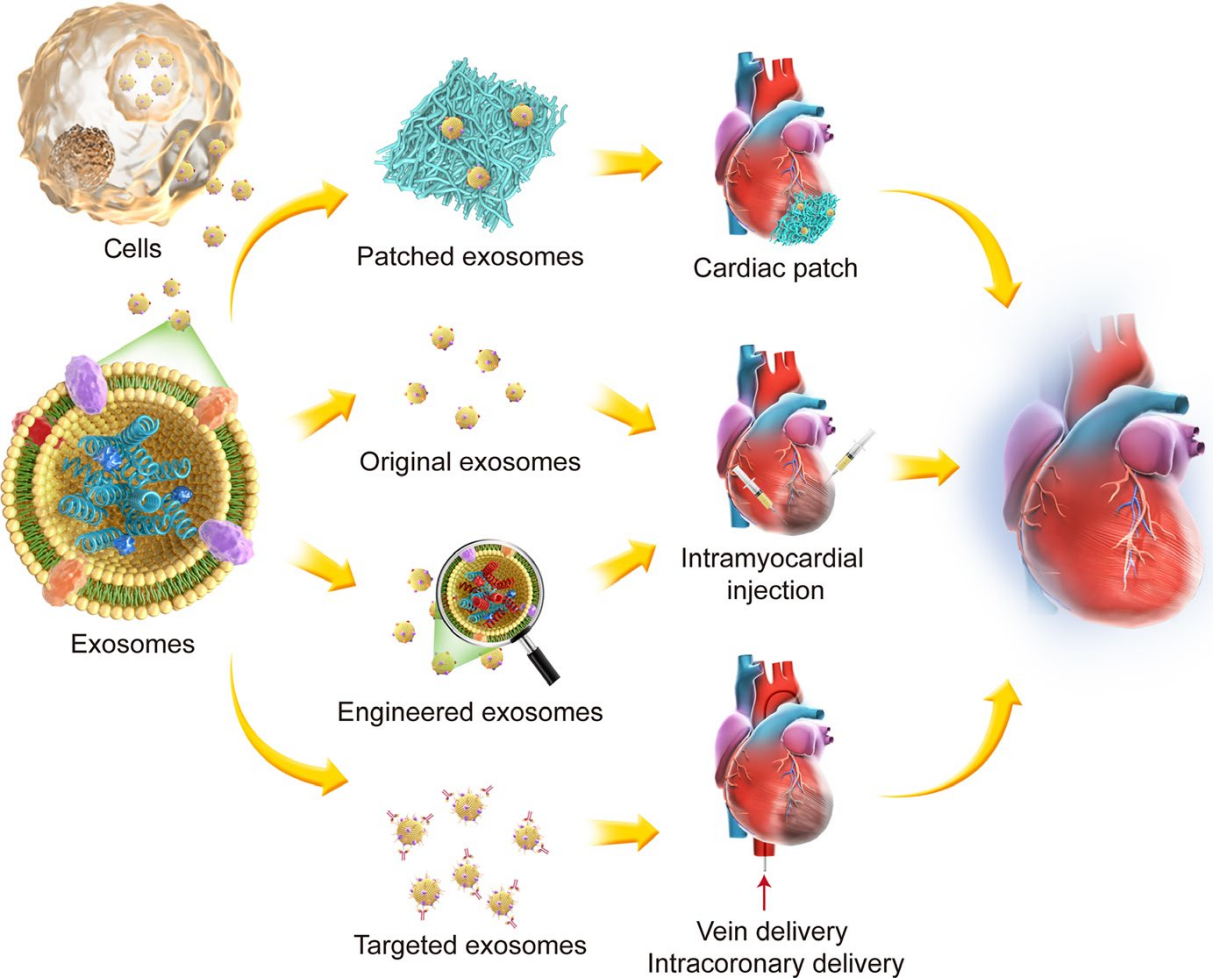
Schematic of tissue engineering application in exosomes treatment. Exosomes themselves or sourced cells can be genetically altered with miRNAs, lncRNAs or tRNAs to express targeted genes using CRISPER/Cas9 or gene delivery method. Exosomes themselves or engineered exosomes could be played as therapeutic vehicles to transport biological substance or chemical compounds and further embedded in scaffolds and delivered as injectable scaffold, patch or 3D tissue construct to enhance the function of exosomes. In addition, exosomes could be engineered with targeted peptides to further improve retention and efficacy when delivery intravenously

Low efficacy due to insufficient exosomes retained at the damaged myocardium ^99^, ^100^ can be one of the major challenges to employ exosome‐based therapy for clinical applications. In addition, exosome‐based therapies still need to address many restrictions, such as uncharacterized off‐target poor effect and purification of complex contents. Although many studies show excellent therapeutic effects of exosomes on CVD models, the methods for delivery to the heart are sub‐optimal. Those delivery methods primarily include systemic injection through the tail vein, intracoronary delivery or intramyocardial injection. Because of its lack of first‐pass effect, intramyocardial delivery is considered to be more effective than the other methods.^101^ However, intramyocardial injection is invasive and infeasible for multiple treatments in current clinical setting. Different delivery strategies have been developed to overcome the limitations as shown in Figure 4. Many new targeting molecules, such as the targeting peptide CSTSMLKAC, have been developed for exosome conjugation,^102^ and these approaches could enhance the retention and achieve target delivery of exosomes for cardiac tissue repair. For example, Vandergriff et al^103^ designed myocardium‐targeting exosomes by reacting DOPE‐NHS with cardiac homing peptide and by inserting the targeting peptide into the exosomal membrane. Additionally, recent advances in biomaterials such as cardiac patches and hydrogels have enabled the delivery of EVs to the heart in a sustained, minimally invasive and slow‐release manner as therapeutic manners to induce endogenous repair.^104^ Gordana Vunjak‐Novakovic and colleagues recently showed that implanted hydrogel patches can deliver purified EVs originating from iCMs to the heart over an extended period of time, and this treatment significantly improved cardiac recovery following ischaemic injury because of the improved retention rate.^105^ Han et al^106^ have encapsulated the human umbilical cord MSC‐derived exosomes in antioxidant peptide, which could enhance therapeutic effects with better target. Nevertheless, the development of targeted exosome delivery approaches with enhanced retention still need to be further explored. Also, those delivery approaches should be able to be incorporated with a minimally invasive surgical approach such as CT or ultrasound guide tube pericardiostomy to reduce the risk associated with the treatment.

The predictability and control over the cargos of exosomes needs to be addressed in order to achieve consistent therapeutic effect. The components of exosomes, namely proteins, lipids, mRNAs and miRNAs, are determined by the source and types of the cells as well as the status of cells during the release process.^107^ Given the exosome‐mediated beneficial effects heavily depend on the content of exosome, it is important to understand the cells that release exosome. For example, MSC‐exosomes are abundant with miR‐21a‐5p, which could down‐regulate the level of targeted pro‐apoptotic genes ^108^; meanwhile, exosomes derived from hypoxia elicited MSCs with high miR‐125b expression primarily reduce cell death to protect heart.^80^ Especially, it has been reported that miR‐106a‐363 cluster was overexpressed in hypoxia iCMs‐derived exosomes when compared to those in normal one.^109^ Therefore, further studies need to acknowledge the pathway to affect the sorting of exosomal cargo, and the fundamental role of various cargo in the therapeutic process in CVDs. In addition, a standard protocol of isolation and purification of exosome should be established to provide a consistent approach to harvest exosomes, with a sophisticated quality control system to ensure the consistence of the components within exosomes for clinical practices.^12^

Exosomes inherit the relative substance from cells under the physiological condition and could be released into tissue space and circulation, and further affect the station of neighbour cells or remote organs. Guay et al^110^ suggested that exosomes could be one novel player in metabolic organ (ie heart, kidney, adipose) crosstalk. Gonzalez‐Calero et al^111^ indicated that exosomes could be a potential key target in cardiorenal syndrome. Valadi et al^97^ advocated that exosomes containing mRNA and miRNA could be delivered to other cells and can work at the new location. Recently, it has been reported that EVs from dysfunctional adipose cells could transfer specific miRNAs and further enhance cardiomyocytes apoptosis through AMPK pathway.^112^ Thus, the miRNAs or other profiles released by cardiac tissue or adipose tissue might have effect on other metabolic organ and even favour the emergence of complications related to CVDs and other metabolism‐related diseases. Although identifying the tissues of origin for circulating EVs may be important in implying the certain relationship to disease, the capacity of exosomes to transfer proteins and RNAs to distant cells suggests their utility as novel drug vehicles and therapeutic targets.

## 7 CONCLUSION

In summary, the promise and excitement surrounding exosomes in cardiovascular research can be manifested daily by newly reported studies. Exosomes may act as messengers and regulate the communication between the heart and other organs, such as the kidney or brain, or the formation of thrombosis in the distal vein. Exosomes might, therefore, offer tools to predict the progression of disease or work as a natural nano‐scale delivery system carrying cargo with a specific, targeted function, or they may serve as therapeutic targets. Although the field of exosomes still has much to be explored, the exploration and specific application of exosomes in cardiovascular disease and potential treatment will continue to be a rapidly advancing focus for cardiovascular researchers.

## CONFLICT OF INTEREST

All authors declare that they have no conflicts of interests.

## AUTHOR CONTRIBUTION

**Jianchao Zhang:** Investigation (equal); Visualization (equal); Writing‐original draft (equal); Writing‐review & editing (equal). **Xiaolin Cui:** Funding acquisition (equal); Investigation (equal); Visualization (equal); Writing‐original draft (equal); Writing‐review & editing (lead). **Jiacheng Guo:** Investigation (equal); Validation (equal); Visualization (supporting); Writing‐review & editing (supporting) **Chang Cao:** Formal analysis (equal); Investigation (equal); Validation (equal). **Zenglei Zhang:** Formal analysis (equal); Investigation (equal); Validation (equal); Visualization (supporting). **Bo Wang:** Formal analysis (equal); Investigation (equal); Validation (equal). **Li Zhang:** Formal analysis (equal); Investigation (equal); Visualization (supporting). **Deliang Shen:** Formal analysis (equal); Investigation (equal); Visualization (supporting). **Khoon Lim:** Investigation (equal); Writing‐review & editing (supporting). **Tim Woodfield:** Writing‐review & editing (supporting). **Junnan Tang:** Conceptualization (lead); Funding acquisition (equal); Investigation (equal); Project administration (lead); Visualization (lead); Writing‐original draft (lead); Writing‐review & editing (lead). **Jinying Zhang:** Conceptualization (equal); Funding acquisition (equal); Project administration (equal); Writing‐review & editing (equal). 

## References

[1] Shpilsky D , Bambs C , Kip K , et al. Association between ideal cardiovascular health and markers of subclinical cardiovascular disease. Clin Cardiol. 2018;41:1593‐1599. 30318617 10.1002/clc.23096 PMC6490110

[2] Zhou M , Wang H , Zeng X , et al. Mortality, morbidity, and risk factors in China and its provinces, 1990–2017: a systematic analysis for the Global Burden of Disease Study 2017. Lancet. 2019;394:1145‐1158. 31248666 10.1016/S0140-6736(19)30427-1 PMC6891889

[3] Tang J , Cui X , Caranasos TG , et al. Heart repair using nanogel‐encapsulated human cardiac stem cells in mice and pigs with myocardial infarction. ACS Nano. 2017;11:9738‐9749. 28929735 10.1021/acsnano.7b01008 PMC5656981

[4] Cui X , Tang J , Hartanto Y , et al. NIPAM‐based microgel microenvironment regulates the therapeutic function of cardiac stromal cells. ACS Appl Mater Interfaces. 2018;10:37783‐37796. 30360109 10.1021/acsami.8b09757 PMC7034655

[5] Tang JN , Cores J , Huang K , et al. Concise review: is cardiac cell therapy dead? Embarrassing trial outcomes and new directions for the future. Stem Cells Transl Med. 2018;7:354‐359. 29468830 10.1002/sctm.17-0196 PMC5866934

[6] Raposo G , Nijman HW , Stoorvogel W , et al. B lymphocytes secrete antigen‐presenting vesicles. J Exp Med. 1996;183:1161‐1172. 8642258 10.1084/jem.183.3.1161 PMC2192324

[7] van Niel G , D'Angelo G , Raposo G . Shedding light on the cell biology of extracellular vesicles. Nat Rev Mol Cell Biol. 2018;19:213‐228. 29339798 10.1038/nrm.2017.125

[8] Turchinovich A , Drapkina O , Tonevitsky A . Transcriptome of extracellular vesicles: state‐of‐the‐art. Front Immunol. 2019;10:202. 30873152 10.3389/fimmu.2019.00202 PMC6404625

[9] Harding C , Heuser J , Stahl P . Receptor‐mediated endocytosis of transferrin and recycling of the transferrin receptor in rat reticulocytes. J Cell Biol. 1983;97:329‐339. 6309857 10.1083/jcb.97.2.329 PMC2112509

[10] Chistiakov DA , Orekhov AN , Bobryshev YV . Cardiac extracellular vesicles in normal and infarcted heart. Int J Mol Sci. 2016;17:63. 10.3390/ijms17010063 PMC4730308 26742038

[11] De Toro J , Herschlik L , Waldner C , Mongini C . Emerging roles of exosomes in normal and pathological conditions: new insights for diagnosis and therapeutic applications. Front Immunol. 2015;6:203. 25999947 10.3389/fimmu.2015.00203 PMC4418172

[12] Pluchino S , Smith JA . Explicating exosomes: reclassifying the rising stars of intercellular communication. Cell. 2019;177:225‐227. 30951665 10.1016/j.cell.2019.03.020

[13] Wiklander O , Brennan MA , Lotvall J , et al. Advances in therapeutic applications of extracellular vesicles. Sci Transl Med. 2019;11:eaav8521. 31092696 10.1126/scitranslmed.aav8521 PMC7104415

[14] O'Loughlin AJ , Woffindale CA , Wood MJ . Exosomes and the emerging field of exosome‐based gene therapy. Curr Gene Ther. 2012;12:262‐274. 22856601 10.2174/156652312802083594

[15] Barile L , Lionetti V , Cervio E , et al. Extracellular vesicles from human cardiac progenitor cells inhibit cardiomyocyte apoptosis and improve cardiac function after myocardial infarction. Cardiovasc Res. 2014;103:530‐541. 25016614 10.1093/cvr/cvu167

[16] Ibrahim AG , Cheng K , Marbán E . Exosomes as critical agents of cardiac regeneration triggered by cell therapy. Stem Cell Reports. 2014;2:606‐619. 24936449 10.1016/j.stemcr.2014.04.006 PMC4050492

[17] Khan M , Nickoloff E , Abramova T , et al. Embryonic stem cell‐derived exosomes promote endogenous repair mechanisms and enhance cardiac function following myocardial infarction. Circ Res. 2015;117:52‐64. 25904597 10.1161/CIRCRESAHA.117.305990 PMC4482130

[18] Huotari J , Helenius A . Endosome maturation. EMBO J. 2011;30:3481‐3500. 21878991 10.1038/emboj.2011.286 PMC3181477

[19] Ostrowski M , Carmo NB , Krumeich S , et al. Rab27a and Rab27b control different steps of the exosome secretion pathway. Nat Cell Biol. 2010;12:19‐30. 19966785 10.1038/ncb2000

[20] Emanueli C , Shearn AI , Angelini GD , et al. Exosomes and exosomal miRNAs in cardiovascular protection and repair. Vascul Pharmacol. 2015;71:24‐30. 25869502 10.1016/j.vph.2015.02.008 PMC4838026

[21] Colombo M , Moita C , van Niel G , et al. Analysis of ESCRT functions in exosome biogenesis, composition and secretion highlights the heterogeneity of extracellular vesicles. J Cell Sci. 2013;126:5553‐5565. 24105262 10.1242/jcs.128868

[22] Hoshino D , Kirkbride KC , Costello K , et al. Exosome secretion is enhanced by invadopodia and drives invasive behavior. Cell Rep. 2013;5:1159‐1168. 24290760 10.1016/j.celrep.2013.10.050 PMC3873329

[23] Hessvik NP , Llorente A . Current knowledge on exosome biogenesis and release. Cell Mol Life Sci. 2018;75:193‐208. 28733901 10.1007/s00018-017-2595-9 PMC5756260

[24] Chairoungdua A , Smith DL , Pochard P , et al. Exosome release of beta‐catenin: a novel mechanism that antagonizes Wnt signaling. J Cell Biol. 2010;190:1079‐1091. 20837771 10.1083/jcb.201002049 PMC3101591

[25] Hurwitz SN , Conlon MM , Rider MA , et al. Nanoparticle analysis sheds budding insights into genetic drivers of extracellular vesicle biogenesis. J Extracell Vesicles. 2016;5:31295. 27421995 10.3402/jev.v5.31295 PMC4947197

[26] Maas S , Breakefield XO , Weaver AM . Extracellular vesicles: unique intercellular delivery vehicles. Trends Cell Biol. 2017;27:172‐188. 27979573 10.1016/j.tcb.2016.11.003 PMC5318253

[27] Simons M , Raposo G . Exosomes–vesicular carriers for intercellular communication. Curr Opin Cell Biol. 2009;21:575‐581. 19442504 10.1016/j.ceb.2009.03.007

[28] Pallet N , Sirois I , Bell C , et al. A comprehensive characterization of membrane vesicles released by autophagic human endothelial cells. Proteomics. 2013;13:1108‐1120. 23436686 10.1002/pmic.201200531

[29] Yáñez‐Mó M , Siljander PR , Andreu Z , et al. Biological properties of extracellular vesicles and their physiological functions. J Extracell Vesicles. 2015;4:27066. 25979354 10.3402/jev.v4.27066 PMC4433489

[30] Crescitelli R , Lasser C , Szabo TG , et al. Distinct RNA profiles in subpopulations of extracellular vesicles: apoptotic bodies, microvesicles and exosomes. J Extracell Vesicles. 2013;2:20677. 10.3402/jev.v2i0.20677 PMC3823106 24223256

[31] Simpson RJ , Lim JW , Moritz RL , et al. Exosomes: proteomic insights and diagnostic potential. Expert Rev Proteomics. 2009;6:267‐283. 19489699 10.1586/epr.09.17

[32] Jeppesen DK , Fenix AM , Franklin JL , et al. Reassessment of exosome composition. Cell. 2019;177:428‐445. 30951670 10.1016/j.cell.2019.02.029 PMC6664447

[33] Brügger B , Bankaitis VA . Lipids and vesicular transport. Biochim Biophys Acta. 2012;1821:1039. 22659299 10.1016/j.bbalip.2012.05.005

[34] Kobayashi T , Gu F , Gruenberg J . Lipids, lipid domains and lipid‐protein interactions in endocytic membrane traffic. Semin Cell Dev Biol. 1998;9:517‐526. 9835639 10.1006/scdb.1998.0257

[35] Subra C , Laulagnier K , Perret B , Record M . Exosome lipidomics unravels lipid sorting at the level of multivesicular bodies. Biochimie. 2007;89:205‐212. 17157973 10.1016/j.biochi.2006.10.014

[36] Ong SG , Lee WH , Huang M , et al. Cross talk of combined gene and cell therapy in ischemic heart disease: role of exosomal microRNA transfer. Circulation. 2014;130:S60‐S69. 25200057 10.1161/CIRCULATIONAHA.113.007917 PMC4862832

[37] Chevillet JR , Kang Q , Ruf IK , et al. Quantitative and stoichiometric analysis of the microRNA content of exosomes. Proc Natl Acad Sci USA. 2014;111:14888‐14893. 25267620 10.1073/pnas.1408301111 PMC4205618

[38] Thery C , Ostrowski M , Segura E . Membrane vesicles as conveyors of immune responses. Nat Rev Immunol. 2009;9:581‐593. 19498381 10.1038/nri2567

[39] Fevrier B , Raposo G . Exosomes: endosomal‐derived vesicles shipping extracellular messages. Curr Opin Cell Biol. 2004;16:415‐421. 15261674 10.1016/j.ceb.2004.06.003

[40] van der Pol E , van Gemert MJ , Sturk A , et al. Single vs. swarm detection of microparticles and exosomes by flow cytometry. J Thromb Haemost. 2012;10:919‐930. 22394434 10.1111/j.1538-7836.2012.04683.x

[41] Sharma S , Rasool HI , Palanisamy V , et al. Structural‐mechanical characterization of nanoparticle exosomes in human saliva, using correlative AFM, FESEM, and force spectroscopy. ACS Nano. 2010;4:1921‐1926. 20218655 10.1021/nn901824n PMC2866049

[42] Tatischeff I , Larquet E , Falcon‐Perez JM , et al. Fast characterisation of cell‐derived extracellular vesicles by nanoparticles tracking analysis, cryo‐electron microscopy, and Raman tweezers microspectroscopy. J Extracell Vesicles. 2012;1:19179. 10.3402/jev.v1i0.19179 PMC3760651 24009887

[43] van der Pol E , Coumans F , Varga Z , et al. Innovation in detection of microparticles and exosomes. J Thromb Haemost. 2013;11:36‐45. 23809109 10.1111/jth.12254

[44] Patil M , Henderson J , Luong H , et al. The art of intercellular wireless communications: exosomes in heart disease and therapy. Front Cell Dev Biol. 2019;7:315. 31850349 10.3389/fcell.2019.00315 PMC6902075

[45] Sluijter JP , Verhage V , Deddens JC , et al. Microvesicles and exosomes for intracardiac communication. Cardiovasc Res. 2014;102:302‐311. 24488559 10.1093/cvr/cvu022

[46] Hergenreider E , Heydt S , Treguer K , et al. Atheroprotective communication between endothelial cells and smooth muscle cells through miRNAs. Nat Cell Biol. 2012;14:249‐256. 22327366 10.1038/ncb2441

[47] Wang C , Zhang C , Liu L , et al. Macrophage‐derived mir‐155‐containing exosomes suppress fibroblast proliferation and promote fibroblast inflammation during cardiac injury. Mol Ther. 2017;25:192‐204. 28129114 10.1016/j.ymthe.2016.09.001 PMC5363311

[48] Gao W , Liu H , Yuan J , et al. Exosomes derived from mature dendritic cells increase endothelial inflammation and atherosclerosis via membrane TNF‐alpha mediated NF‐kappaB pathway. J Cell Mol Med. 2016;20:2318‐2327. 27515767 10.1111/jcmm.12923 PMC5134386

[49] Yu X , Deng L , Wang D , et al. Mechanism of TNF‐alpha autocrine effects in hypoxic cardiomyocytes: initiated by hypoxia inducible factor 1alpha, presented by exosomes. J Mol Cell Cardiol. 2012;53:848‐857. 23085511 10.1016/j.yjmcc.2012.10.002

[50] Louapre P , Grongnet JF , Tanguay RM , et al. Effects of hypoxia on stress proteins in the piglet heart at birth. Cell Stress Chaperones. 2005;10:17‐23. 15832944 10.1379/CSC-74R.1 PMC1074566

[51] Boulanger CM , Scoazec A , Ebrahimian T , et al. Circulating microparticles from patients with myocardial infarction cause endothelial dysfunction. Circulation. 2001;104:2649‐2652. 11723013 10.1161/hc4701.100516

[52] Deng ZB , Poliakov A , Hardy RW , et al. Adipose tissue exosome‐like vesicles mediate activation of macrophage‐induced insulin resistance. Diabetes. 2009;58:2498‐2505. 19675137 10.2337/db09-0216 PMC2768161

[53] Mesri M , Altieri DC . Endothelial cell activation by leukocyte microparticles. J Immunol. 1998;161:4382‐4387. 9780216

[54] Martin S , Tesse A , Hugel B , et al. Shed membrane particles from T lymphocytes impair endothelial function and regulate endothelial protein expression. Circulation. 2004;109:1653‐1659. 15023873 10.1161/01.CIR.0000124065.31211.6E

[55] Ruparelia N , Chai JT , Fisher EA , et al. Inflammatory processes in cardiovascular disease: a route to targeted therapies. Nat Rev Cardiol. 2017;14:133‐144. 27905474 10.1038/nrcardio.2016.185 PMC5525550

[56] Lee SW , Won JY , Kim WJ , et al. Snail as a potential target molecule in cardiac fibrosis: paracrine action of endothelial cells on fibroblasts through snail and CTGF axis. Mol Ther. 2013;21:1767‐1777. 23760445 10.1038/mt.2013.146 PMC3953993

[57] Qiao L , Hu S , Liu S , et al. microRNA‐21‐5p dysregulation in exosomes derived from heart failure patients impairs regenerative potential. J Clin Invest. 2019;129:2237‐2250. 31033484 10.1172/JCI123135 PMC6546482

[58] de Couto G , Gallet R , Cambier L , et al. Exosomal microRNA transfer into macrophages mediates cellular postconditioning. Circulation. 2017;136:200‐214. 28411247 10.1161/CIRCULATIONAHA.116.024590 PMC5505791

[59] Cambier L , de Couto G , Ibrahim A , et al. Y RNA fragment in extracellular vesicles confers cardioprotection via modulation of IL‐10 expression and secretion. EMBO Mol Med. 2017;9:337‐352. 28167565 10.15252/emmm.201606924 PMC5331234

[60] Cheng M , Yang J , Zhao X , et al. Circulating myocardial microRNAs from infarcted hearts are carried in exosomes and mobilise bone marrow progenitor cells. Nat Commun. 2019;10:1‐9. 30814518 10.1038/s41467-019-08895-7 PMC6393447

[61] Oikonomou EK , Antoniades C . The role of adipose tissue in cardiovascular health and disease. Nat Rev Cardiol. 2019;16:83‐99. 30287946 10.1038/s41569-018-0097-6

[62] Xie Z , Wang X , Liu X , et al. Adipose‐derived exosomes exert proatherogenic effects by regulating macrophage foam cell formation and polarization. J Am Heart Assoc. 2018;7:e007442. 29502100 10.1161/JAHA.117.007442 PMC5866320

[63] Wang X , Gu H , Huang W , et al. Hsp20‐mediated activation of exosome biogenesis in cardiomyocytes improves cardiac function and angiogenesis in diabetic mice. Diabetes. 2016;65:3111‐3128. 27284111 10.2337/db15-1563 PMC5033265

[64] Sluijter J , Davidson SM , Boulanger CM , et al. Extracellular vesicles in diagnostics and therapy of the ischaemic heart: Position Paper from the Working Group on Cellular Biology of the Heart of the European Society of Cardiology. Cardiovasc Res. 2018;114:19‐34. 29106545 10.1093/cvr/cvx211 PMC5852624

[65] Lawson C , Vicencio JM , Yellon DM , et al. Microvesicles and exosomes: new players in metabolic and cardiovascular disease. J Endocrinol. 2016;228:R57‐71. 26743452 10.1530/JOE-15-0201

[66] Kuwabara Y , Ono K , Horie T , et al. Increased microRNA‐1 and microRNA‐133a levels in serum of patients with cardiovascular disease indicate myocardial damage. Circ Cardiovasc Genet. 2011;4:446‐454. 21642241 10.1161/CIRCGENETICS.110.958975

[67] Cheow ES , Cheng WC , Lee CN , et al. Plasma‐derived extracellular vesicles contain predictive biomarkers and potential therapeutic targets for myocardial ischemic (MI) injury. Mol Cell Proteomics. 2016;15:2628‐2640. 27234505 10.1074/mcp.M115.055731 PMC4974341

[68] Vegter EL , van der Meer P , de Windt LJ , et al. MicroRNAs in heart failure: from biomarker to target for therapy. Eur J Heart Fail. 2016;18:457‐468. 26869172 10.1002/ejhf.495

[69] Fleissner F , Goerzig Y , Haverich A , et al. Microvesicles as novel biomarkers and therapeutic targets in transplantation medicine. Am J Transplant. 2012;12:289‐297. 22082333 10.1111/j.1600-6143.2011.03790.x

[70] Tian C , Gao L , Zimmerman MC , Zucker IH . Myocardial infarction‐induced microRNA‐enriched exosomes contribute to cardiac Nrf2 dysregulation in chronic heart failure. Am J Physiol Heart Circ Physiol. 2018;314:928‐939. 10.1152/ajpheart.00602.2017 PMC6008149 29373037

[71] Gupta S , Knowlton AA . HSP60 trafficking in adult cardiac myocytes: role of the exosomal pathway. Am J Physiol Heart Circ Physiol. 2007;292:3052‐3056. 10.1152/ajpheart.01355.2006 17307989

[72] Goren Y , Kushnir M , Zafrir B , et al. Serum levels of microRNAs in patients with heart failure. Eur J Heart Fail. 2012;14:147‐154. 22120965 10.1093/eurjhf/hfr155

[73] Creemers EE , Tijsen AJ , Pinto YM . Circulating microRNAs: novel biomarkers and extracellular communicators in cardiovascular disease? Circ Res. 2012;110:483‐495. 22302755 10.1161/CIRCRESAHA.111.247452

[74] Duan P , Tan J , Miao Y , et al. Potential role of exosomes in the pathophysiology, diagnosis, and treatment of hypoxic diseases. Am J Transl Res. 2019;11:1184‐1201. 30972155 PMC6456517

[75] Heallen TR , Kadow ZA , Kim JH , et al. Stimulating cardiogenesis as a treatment for heart failure. Circ Res. 2019;124:1647‐1657. 31120819 10.1161/CIRCRESAHA.118.313573 PMC6534162

[76] Adamiak M , Sahoo S . Exosomes in myocardial repair: advances and challenges in the development of next‐generation therapeutics. Mol Ther. 2018;26:1635‐1643. 29807783 10.1016/j.ymthe.2018.04.024 PMC6035738

[77] Singla DK . Stem cells and exosomes in cardiac repair. Curr Opin Pharmacol. 2016;27:19‐23. 26848944 10.1016/j.coph.2016.01.003

[78] Lamichhane TN , Sokic S , Schardt JS , et al. Emerging roles for extracellular vesicles in tissue engineering and regenerative medicine. Tissue Eng Part B Rev. 2015;21:45‐54. 24957510 10.1089/ten.teb.2014.0300 PMC4321981

[79] Bjørge IM , Kim SY , Mano JF , et al. Extracellular vesicles, exosomes and shedding vesicles in regenerative medicine – a new paradigm for tissue repair. Biomater Sci. 2017;6:60‐78. 29184934 10.1039/c7bm00479f

[80] Zhu LP , Tian T , Wang JY , et al. Hypoxia‐elicited mesenchymal stem cell‐derived exosomes facilitates cardiac repair through miR‐125b‐mediated prevention of cell death in myocardial infarction. Theranostics. 2018;8:6163‐6177. 30613290 10.7150/thno.28021 PMC6299684

[81] Lai RC , Arslan F , Lee MM , et al. Exosome secreted by MSC reduces myocardial ischemia/reperfusion injury. Stem Cell Res. 2010;4:214‐222. 20138817 10.1016/j.scr.2009.12.003

[82] Gallet R , Dawkins J , Valle J , et al. Exosomes secreted by cardiosphere‐derived cells reduce scarring, attenuate adverse remodelling, and improve function in acute and chronic porcine myocardial infarction. Eur Heart J. 2017;38:201‐211. 28158410 10.1093/eurheartj/ehw240 PMC5837390

[83] Nguyen CT , Dawkins J , Bi X , et al. Diffusion tensor cardiac magnetic resonance reveals exosomes from cardiosphere‐derived cells preserve myocardial fiber architecture after myocardial infarction. JACC Basic Transl Sci. 2018;3:97‐109. 29600288 10.1016/j.jacbts.2017.09.005 PMC5869026

[84] Ciullo A , Biemmi V , Milano G , et al. Exosomal expression of CXCR4 targets cardioprotective vesicles to myocardial infarction and improves outcome after systemic administration. Int J Mol Sci. 2019;20:468. 10.3390/ijms20030468 PMC6386845 30678240

[85] Barile L , Cervio E , Lionetti V , et al. Cardioprotection by cardiac progenitor cell‐secreted exosomes: role of pregnancy‐associated plasma protein‐A. Cardiovasc Res. 2018;114:992‐1005. 29518183 10.1093/cvr/cvy055

[86] Jung JH , Fu X , Yang PC . Exosomes generated from iPSC‐derivatives: new direction for stem cell therapy in human heart diseases. Circ Res. 2017;120:407‐417. 28104773 10.1161/CIRCRESAHA.116.309307 PMC5260934

[87] Adamiak M , Cheng G , Bobis‐Wozowicz S , et al. Induced pluripotent stem cell (iPSC)‐derived extracellular vesicles are safer and more effective for cardiac repair than iPSCs. Circ Res. 2018;122:296‐309. 29118058 10.1161/CIRCRESAHA.117.311769 PMC5775034

[88] Mayourian J , Ceholski DK , Gorski PA , et al. Exosomal microRNA‐21‐5p mediates mesenchymal stem cell paracrine effects on human cardiac tissue contractility. Circ Res. 2018;122:933‐944. 29449318 10.1161/CIRCRESAHA.118.312420 PMC5986183

[89] Marote A , Teixeira FG , Mendes‐Pinheiro B , et al. MSCs‐derived exosomes: cell‐secreted nanovesicles with regenerative potential. Front Pharmacol. 2016;7:231. 27536241 10.3389/fphar.2016.00231 PMC4971062

[90] Sahoo S , Klychko E , Thorne T , et al. Exosomes from human CD34(+) stem cells mediate their proangiogenic paracrine activity. Circ Res. 2011;109:724‐728. 21835908 10.1161/CIRCRESAHA.111.253286 PMC3201702

[91] Mathiyalagan P , Liang Y , Kim D , et al. Angiogenic mechanisms of human CD34(+) stem cell exosomes in the repair of ischemic hindlimb. Circ Res. 2017;120:1466‐1476. 28298297 10.1161/CIRCRESAHA.116.310557 PMC5420547

[92] Teng X , Chen L , Chen W , et al. Mesenchymal stem cell‐derived exosomes improve the microenvironment of infarcted myocardium contributing to angiogenesis and anti‐inflammation. Cell Physiol Biochem. 2015;37:2415‐2424. 26646808 10.1159/000438594

[93] Zhao J , Li X , Hu J , et al. Mesenchymal stromal cell‐derived exosomes attenuate myocardial ischaemia‐reperfusion injury through miR‐182‐regulated macrophage polarization. Cardiovasc Res. 2019;115:1205‐1216. 30753344 10.1093/cvr/cvz040 PMC6529919

[94] Zhang Z , Yang J , Yan W , et al. Pretreatment of cardiac stem cells with exosomes derived from mesenchymal stem cells enhances myocardial repair. J Am Heart Assoc. 2016;5:e002856. 26811168 10.1161/JAHA.115.002856 PMC4859399

[95] Foglio E , Puddighinu G , Fasanaro P , et al. Exosomal clusterin, identified in the pericardial fluid, improves myocardial performance following MI through epicardial activation, enhanced arteriogenesis and reduced apoptosis. Int J Cardiol. 2015;197:333‐347. 26159041 10.1016/j.ijcard.2015.06.008

[96] Biemmi V , Milano G , Ciullo A , et al. Inflammatory extracellular vesicles prompt heart dysfunction via TRL4‐dependent NF‐κB activation. Theranostics. 2020;10:2773‐2790. 32194834 10.7150/thno.39072 PMC7052909

[97] Valadi H , Ekstrom K , Bossios A , et al. Exosome‐mediated transfer of mRNAs and microRNAs is a novel mechanism of genetic exchange between cells. Nat Cell Biol. 2007;9:654‐659. 17486113 10.1038/ncb1596

[98] Sluijter JP , van Rooij E . Exosomal microRNA clusters are important for the therapeutic effect of cardiac progenitor cells. Circ Res. 2015;116:219‐221. 25593269 10.1161/CIRCRESAHA.114.305673

[99] Le Bras A . Exosome‐based therapy to repair the injured heart. Nat Rev Cardiol. 2018;15:382. 10.1038/s41569-018-0027-7 29748593

[100] Gurunathan S , Kang MH , Jeyaraj M , et al. Review of the isolation, characterization, biological function, and multifarious therapeutic approaches of exosomes. Cells. 2019;8:307. 10.3390/cells8040307 PMC6523673 30987213

[101] Takahashi Y , Nishikawa M , Shinotsuka H , et al. Visualization and in vivo tracking of the exosomes of murine melanoma B16‐BL6 cells in mice after intravenous injection. J Biotechnol. 2013;165:77‐84. 23562828 10.1016/j.jbiotec.2013.03.013

[102] Wang X , Chen Y , Zhao Z , et al. Engineered exosomes with ischemic myocardium‐targeting peptide for targeted therapy in myocardial infarction. J Am Heart Assoc. 2018;7:e008737. 30371236 10.1161/JAHA.118.008737 PMC6201471

[103] Vandergriff A , Huang K , Shen D , et al. Targeting regenerative exosomes to myocardial infarction using cardiac homing peptide. Theranostics. 2018;8:1869‐1878. 29556361 10.7150/thno.20524 PMC5858505

[104] Heallen TR , Martin JF . Heart repair via cardiomyocyte‐secreted vesicles. Nat Biomed Eng. 2018;2:271‐272. 30936454 10.1038/s41551-018-0239-5

[105] Liu B , Lee BW , Nakanishi K , et al. Cardiac recovery via extended cell‐free delivery of extracellular vesicles secreted by cardiomyocytes derived from induced pluripotent stem cells. Nat Biomed Eng. 2018;2:293‐303. 30271672 10.1038/s41551-018-0229-7 PMC6159913

[106] Han C , Zhou J , Liang C , et al. Human umbilical cord mesenchymal stem cell derived exosomes encapsulated in functional peptide hydrogels promote cardiac repair. Biomater Sci. 2019;7:2920‐2933. 31090763 10.1039/c9bm00101h

[107] Gartz M , Strande JL . Examining the paracrine effects of exosomes in cardiovascular disease and repair. J Am Heart Assoc. 2018;7:e007954. 29858362 10.1161/JAHA.117.007954 PMC6015376

[108] Luther KM , Haar L , McGuinness M , et al. Exosomal miR‐21a‐5p mediates cardioprotection by mesenchymal stem cells. J Mol Cell Cardiol. 2018;119:125‐137. 29698635 10.1016/j.yjmcc.2018.04.012

[109] Jung JH , Tada Y , Bornstaedt D , et al. Exosomal miR‐106a‐363 cluster from the hypoxic human ipsc‐derived cardiomyocytes restore the ischemic myocardium. J Am Coll Cardiol. 2018;71:A14.

[110] Guay C , Regazzi R . Exosomes as new players in metabolic organ cross‐talk. Diabetes Obes Metab. 2017;19:137‐146. 28880477 10.1111/dom.13027

[111] Gonzalez‐Calero L , Martin‐Lorenzo M , Alvarez‐Llamas G . Exosomes: a potential key target in cardio‐renal syndrome. Front Immunol. 2014;5:465. 25339951 10.3389/fimmu.2014.00465 PMC4189416

[112] Gan L , Xie D , Liu J , et al. Small extracellular microvesicles mediated pathological communications between dysfunctional adipocytes and cardiomyocytes as a novel mechanisms exacerbating ischemia/reperfusion injury in diabetic mice. Circulation. 2020;141:968‐983. 31918577 10.1161/CIRCULATIONAHA.119.042640 PMC7093230

